# Multicentre evaluation of the BYG Carba v2.0 test, a simplified electrochemical assay for the rapid laboratory detection of carbapenemase-producing Enterobacteriaceae

**DOI:** 10.1038/s41598-017-09820-y

**Published:** 2017-08-30

**Authors:** Pierre Bogaerts, Saoussen Oueslati, Danièle Meunier, Claire Nonhoff, Sami Yunus, Marion Massart, Olivier Denis, Neil Woodford, Katie L. Hopkins, Thierry Naas, Laurent Dortet, Te-Din Huang, Youri Glupczynski

**Affiliations:** 1 Laboratory of clinical microbiology, National reference center for monitoring antimicrobial resistance in Gram-negative bacteria, CHU UCL Namur, Yvoir, Belgium; 2 Bacteriology-Hygiene unit, Assistance Publique/Hôpitaux de Paris, Bicêtre Hospital, Le Kremlin-Bicêtre, France; 3 EA7361 “Structure, dynamic, function and expression of broad spectrum β-lactamases”, Université Paris Sud, Université Paris Saclay, LabEx Lermit, Faculty of Medicine, Le Kremlin-Bicêtre, France; 4 Associated French National Reference Center for Antibiotic Resistance: Carbapenemase-producing Enterobacteriaceae, Le Kremlin-Bicêtre, France; 5 Antimicrobial Resistance and Healthcare Associated Infections (AMRHAI) Reference Unit, National Infection Service, Public Health England, London, NW9 5EQ UK; 6 Department of Microbiology, Associated national reference center, Hôpital Erasme, Université Libre de Bruxelles, Route de Lennik 808, 1070 Brussels, Belgium; 7 Bio and Soft Matter, Institute of Condensed Matter and Nanosciences, Université catholique de Louvain, Louvain-La-Neuve, Belgium

## Abstract

Rapid detection of carbapenemase-producing Enterobacteriaceae (CPE) represents a major challenge for microbiology laboratories. We evaluated the BYG Carba v2.0 using a simplified protocol, which detects CPE in less than 30 minutes. This new procedure reduces the hands-on-time from 5 to one minute and only requires a limited amount of material (one to three colonies) thereby preventing the need for subculturing bacterial isolates to reach a larger amount of pure biomass. This multicentre study involved four European reference laboratories. For the 1181 isolates tested across four centres, BYG Carba v2.0 yielded overall sensitivity and specificity of 96.3% (CI95: 94.5–97.5) and 99.7% (CI95: 98.6–100) respectively. Considering only the 670 consecutive isolates tested prospectively, the BYG Carba v2.0 displayed overall positive and negative predictive values of 99.7% (CI95: 95.4–98.9) and 97.5% (CI95: 94.9–98.8). Regarding time to positivity, 85% of CPE detected were positive within ten minutes. The BYG Carba v2.0 is a new highly simplified, rapid and accurate electrochemical assay discriminating between CPE and non-CPE in less than 30 min. The real-time quantified signal allows objective and traceable interpretation of the results.

## Introduction

The emergence and worldwide spread of carbapenemase-producing Enterobacteriaceae (CPE) represents a major public health concern. Accurate and timely detection of CPE is essential for patient management and for rapid implementation of infection control measures^1, 2^.

Various confirmatory tests for the non-molecular detection of CPE have been proposed^3^. The most rapid methods rely on the detection of carbapenem hydrolysis by colorimetric assay or by mass spectrometry^3^. We recently evaluated three colorimetric assays, the RAPIDEC^®^ CARBA NP test (BioMérieux, Marcy l’Etoile, France), the Neo-Rapid CARB kit (Rosco Diagnostica, Taastrup, Denmark) and the β-CARBA^™^ test (Bio-Rad, Marnes-la-Coquette, France) and found that overall all these tests showed satisfactory results for the detection of CPE despite differences in performance between the tests^4^. Nevertheless, all the colorimetric assays are based on a subjective visual observation of colour change, which can be sometimes challenging to interpret, especially for some carbapenemase families (e.g: OXA-48 like, GES-like) with lower hydrolytic activity. Furthermore, the format of the colorimetric assays does not lend itself to automated traceability in a laboratory information system.

We recently developed and evaluated the BYG Carba test, an electrochemical assay that allows the rapid (within 30 minutes) and objective confirmation of carbapenemase activity in Enterobacteriaceae^5^. The BYG Carba test detects variations of conductivity of an electrode coated with polyaniline, an electrosensing polymer. The polyaniline is highly sensitive to the modifications of pH and redox activity, which occur during the hydrolysis of imipenem by a carbapenemase. These modifications, which can be measured and monitored in real-time by the BYG Carba test, are indicative of the presence of an enzyme having carbapenem hydrolytic activity including the five major carbapenemase families (VIM, NDM, IMP, KPC, OXA-48)^5^. The major drawback of this method was that in its original format (BYG v1.0) a heavy bacterial suspension (McFarland 4 corresponding to + /− 10^9^ CFU/ml) was required like for the other colorimetric assays. For the present study, we adapted and modified the BYG assay to use a smaller inoculum corresponding to only one to three colonies (i.e. corresponding approximately to 10^6^ CFU/ml) directly deposited on the electrode (hands-on-time of about one minute). This reduced bacterial load still permits the use of the primary culture plate and avoids the need for additional subculture. The BYG Carba test using this new protocol (v2.0) (supplemental video) was validated in a multicentre survey that was conducted in four laboratories with recognized expertise in the characterization of the mechanisms of antimicrobial resistance (two laboratories in Belgium, one in France and one in the United Kingdom).

## Results

### Preliminary assessment of BYG Carba v2.0 compared to BYG Carba v1.0

A signal cut-off of 3.5 and of 11.5 (arbitrary units [AU]) was defined as the threshold for discrimination between carbapenemase and non-carbapenemase producers for the BYG v1.0 and BYG v2.0, respectively and were established previously using ROC curve^4, 5^.

**Figure 1:**
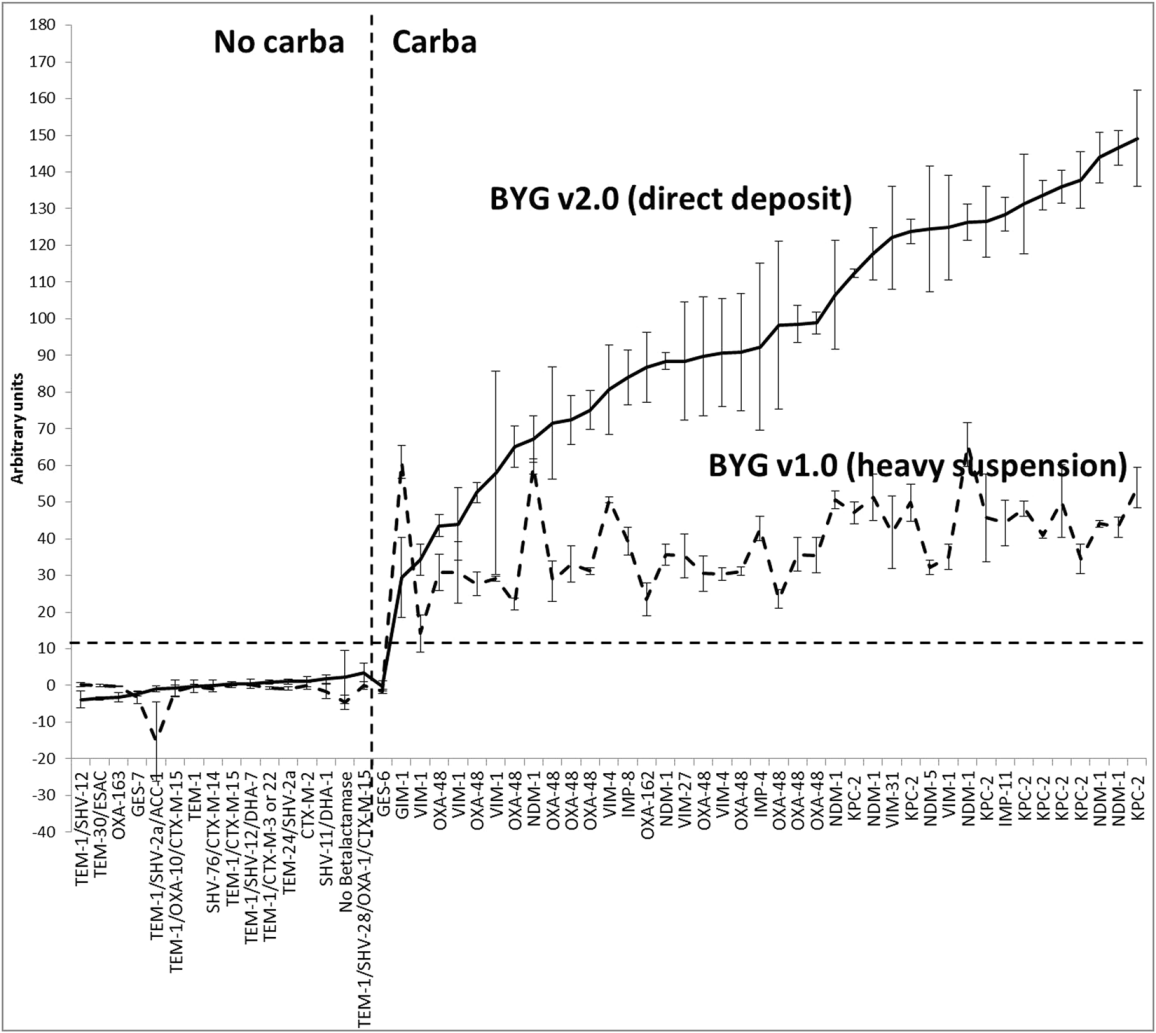
Comparison of polyaniline conductance signals by BYG Carba v1.0 and BYG Carba v2.0 assays. Intensity of the signal is expressed in arbitrary units and reflects the conductance of the sensor (Y axis). Main resistance mechanisms are indicated on the X axis. No carba/Carba vertical dotted line represents the boundary between the non-carbapenemase producers and the carbapenemase producers. Horizontal dotted line is the cut-off of positivity of the test (11.5 AU); Vertical bars on the curves represent standard deviation of 3 independent measures. The mean value for all positive values is 44.0 (CI95: 40.0–48.0) and 97.8 (CI95: 88.0–107.7) for BYG Carba v1.0 and BYG Carba v2.0 respectively and are significantly different (p < 0.00001 by independent two-sample Student’s t-test).

The comparison of the results obtained with BYG v1.0 and BYG v2.0 are presented in Fig. 1. All CPE isolates except one *Citrobacter braakii* isolate producing GES-6, a very weak and rarely reported class A carbapenemase^6^, were correctly identified by the BYG Carba v2.0. GES-6 was also not detected by the BYG Carba v1.0. The maximum value obtained with BYG Carba v2.0 for the non-CPE was 3.5 AU while the minimum value obtained for the CPE detected (GES-6 excluded) was 29.4 AU. The signal intensity of the positive strains when tested in triplicate was significantly higher (p < 0.00001) with BYG Carba v2.0 (Mean = 97.8 AU, CI 95 = 87.9–107.7; Median = 98.5) than with BYG Carba v1.0 (Mean = 44.0 AU, CI 95 = 40.0–48.0; Median = 48.0) with accuracy of CPE detection remaining unchanged for this isolate panel. For the negative results, the mean and median values were not significantly different between BYG Carba v1.0 (Mean = −0.9; Median = −0.1) and BYG Carba v2.0 (Mean = −0.2; median = −0.1).

### BYG Carba v2.0 results on retrospective and prospective clinical isolates

A total of 1181 clinical Enterobacteriaceae isolates were included in this evaluation (511 clinical isolates retrieved from local archives with previously characterized β-lactam resistance mechanisms and 670 consecutive Enterobacteriaceae clinical isolates collected prospectively at the four laboratories; Table 1). According to the reference methods used in each laboratory, this study included 704 CPE (OXA-48-like [n = 359], KPC [n = 114], NDM [n = 107], VIM [n = 78], IMP [n = 17], NDM + OXA-48-like [n = 14] and other miscellaneous carbapenemase-producing Enterobacteriaceae (IMI, SME, NMC-A, GES, FRI and GIM carbapenemases; [n = 15]) as well as 477 non-CPE isolates (Table 2). Among the 1181 tested isolates the species distribution was as following: *Klebsiella pneumoniae* (n = 507), *Escherichia coli* (n = 228), *Enterobacter cloacae* complex (n = 228), *Citrobacter freundii* (n = 59), *Klebsiella oxytoca* (n = 57), *Enterobacter aerogenes* (n = 49), *Serratia marcescens* (n = 20), and miscellaneous others (n = 33). The performance of the BYG Carba v2.0 assay for CPE detection did not differ by bacterial species and was not related to the MICs to carbapenems as had already been observed for BYG Carba assay v1.0^5^.

**Table 1:** Number of clinical isolates analyzed in each centre.

	Retrospective collection (n = 511)	Prospective collection (n = 670)
Centre A	150	258
Centre B	87	111
Centre C	175	201
Centre D	99	100

**Table 2:** BYG Carba v2.0 results for the 1181 clinical isolates analyzed in this study.

	Retrospective collection (n = 511)		Prospective collection (n = 670)	
	Reference method	Detected by BYG Carba (% of correct results)	Reference method	Detected by BYG Carba (% of correct results)
**Total CPE**	**348**	**330 (94.8)**	**356**	**348 (97.8)**
OXA-48-like	127	122 (96.1)	232	227 (97.8)
KPC	71	71 (100)	43	43 (100)
NDM	60	56 (93.3)	47	46 (97.9)
VIM	52	51 (98.1)	26	24 (92.3)
IMP	15	13 (86.7)	2	2 (100)
OXA-48-like + NDM	8	8 (100)	6	6 (100)
IMI	6	4 (66.7)	0	NA
SME	2	2 (100)	0	NA
NMC-A	1	1 (100)	0	NA
GES-5	4	0 (0)	0	NA
FRI-1	1	1 (100)	0	NA
GIM-1	1	1 (100)	0	NA
**Total of Non-CPE**	163*	**0 (100**)	**314**	**1 (99.7)**

*Including 3 isolates expressing an OXA-48 variant devoid of carbapenemase activity (OXA-163 [n = 2] and OXA-405 [n = 1]).

NA: not applicable.

When analyzing separately the subset of archived isolates with known carbapenem resistance mechanisms, the BYG Carba v2.0 correctly identified 330/348 (94.8%) CPE isolates and 163/163 non-CPE isolates including OXA-163 (n = 2) and OXA-405 (n = 1)-producing isolates, two OXA-48 variants lacking any carbapenemase activity (sensitivity of 94.8%, specificity of 100%). When considering the performance of the test for detecting individual carbapenemase families, the BYG Carba v2.0 detected isolates belonging to the five major carbapenemase families with a sensitivity ranging from 86.7% for IMP to 100% for KPC (Table 3). Notably, 122/127 (96.1%) OXA-48-like producing organisms were correctly detected by the BYG Carba v2.0 as were also less common carbapenemase types such as SME, NMC-A, FRI-1and GIM-1. None of the four GES-5-producing isolates were detected by the BYG Carba v2.0 (Table 2).

**Table 3:** Performance of the BYG Carba v2.0 according to carbapenemase family.

	Retrospectively (n = 511) Spec: **100% (CI95: 97.1–100)**	Prospectively (n = 670) Spec: **99.7% (CI95: 97.9–100)**			Total (n = 1181) Spec: **99.7% (CI95: 98.6–100)**
	Sens (%) (CI95)	Sens (%) (CI95)	PPV (%) (CI95)	NPV (%) (CI95)	Sens (%) (CI95)
**CPE**	**94.8 (91.8–96.8)**	**97.7 (95.4–98.9)**	**99.7 (98.2–100)**	**97.5 (94.9–98.8)**	**96.3 (94.5–97.5)**
OXA-48-like	96.1 (90.6–98.5)	97.8 (94.8–99.2)	99.6 (97.2–100)	98.4 (96.1–99.4)	97.2 (94.8–98.6)
KPC	100 (93.6–100)	100 (89.8–100)	97.7 (86.5–99.9)	100 (98.5–100)	100 (95.9–100)
NDM	93.3 (83.0–97.8)	97.9 (87.3–99.9)	97.9 (87.3–99.9)	99.7 (97.9–100)	95.3 (88.9–98.2)
VIM	98.1 (88.4–99.9)	92.3 (73.4–98.6)	96.0 (77.7–99.8)	99.4 (97.4–100)	96.1 (88.4–99.0)
IMP	86.7 (58.4–97.6)	100 (19.8–100)	66.7 (12.5–98.2)	100 (98.5–100)	88.2 (62.2–97.9)
OXA-48-like + NDM	100 (59.8–100)	100 (51.7–100)	85.7 (42.0–99.2)	100 (98.5–100)	100 (73.2–100)

The values are based on the results reported by each centre without any correction of the discrepancies by the CHU UCL Namur.

Sens: sensitivity; Spec: specificity; PPV: positive predictive value; NPV: negative predictive value.

In the prospective part of this evaluation (670 consecutive isolates), the BYG Carba v2.0 detected 348/356 (97.8%) of the CPE while 313/314 (99.7%) (Table 2) of the non-CPE were correctly identified. Across the four centres the performance of the BYG Carba v2.0 was 97.7% (CI95 = 95.4–98.9) sensitivity, 99.7% (CI95 = 97.9–100) specificity, 99.7% (CI95 = 98.2–100) positive predictive value (PPV) and 97.5% (CI95 = 94.9–98.8) negative predictive value (NPV) (Table 3).

Across the four centres out of the total of 1181 isolates, the test showed 96.3% sensitivity and 99.7% specificity (Tables 2 and 4). Overall, only 27 discrepant results were observed (2.3% of the 1181 tested isolates). Fourteen discrepant results were observed in centre D, nine in centre C, two in centre A and two in centre B. Among these, 26 strains yielded a false-negative result (18/511 [3.5%] from the archived isolates and 8/670 [1.2%] from the prospective evaluation) and one false-positive result was observed during the prospective evaluation. The BYG Carba v2.0 failed to detect ten OXA-48-like (OXA-244 [n = 4], OXA-48 [n = 4], OXA-232 and OXA-181 [n = 1, each]), NDM (n = 5), GES carbapenemase variants (n = 4), VIM (n = 3), IMP and IMI-producing Enterobacteriaceae (n = 2, each). The observed false negative values ranged between 5 AU for an IMI-1- producing *E. cloacae* to 11 AU for an IMI-2-producing *E. cloacae*. The false-positive result was detected for a SHV-12-expressing *E. cloacae* with a value of 15 AU (Table 4).

**Table 4:** Signal value obtained with the BYG Carba v2.0 and time to detection for the ‘big five’ carbapenemases.

Type	Total detected	Mean of signal in AU (CI95)	% detected in ≤5 min (CI95)	% detected in ≤10 min (CI95)
CPE	678	97.3 (94.9–99.8)	45.1 (41.4–48.9)	85.2 (82.6–87.9)
OXA-48-like	349	85.3 (82.3–88.3)	43.8 (38.6–49.0)	89.1 (85.8–92.4)
KPC	114	122.2 (117.4–127.1)	75.4 (67.5–83.3)	94.7 (90.6–98.8)
NDM	102	114.7 (109.1–120.2)	54.9 (45.2–64.6)	90.2 (84.4–96.0)
VIM	75	99.3 (92.0–106.6)	8 (3.7–17.7)	54.7 (43.4–65.9)
IMP	15	72.5 (54.9–90.0)	0	53.3 (28.1–78.5)

AU: Arbitrary units.

CI: confidence interval.

The 27 strains that did not yield matching results were revaluated in centre A for possible resolution of the discrepancies and repeat screening with the BYG test. False-negative results were confirmed for 17 strains while for 10 strains, the repeated results were in agreement with the results of the molecular tests obtained in centre A, therefore suggesting a loss of plasmid or a technical problem in the original testing centre (Table 5). In particular, the false-positive *E. cloacae* was repeatedly negative when tested 3 times (true-negative), one NDM-1 and one OXA-48-producing strains were phenotypically fully susceptible when received in center A (suggesting loss of plasmid) and seven strains were retested positive in center A (GES-5, IMI-1, IMP-1, NDM-1, OXA-244 and OXA-48, [n = 1] each). In addition, these discrepant isolates were also tested with the Carba NP test which showed 13 positive results.This suggests that the carbapenemase activity of the 14 negative isolates is particularly low (either because of a lack of expression of the carbapenemase or because of a low carbapenemase activity of the variant [for example OXA-244]). The time to positivity was also determined along with the value of the signal at the end of the run. For the 678 out of 704 CPE (96.3%) detected by the BYG Carba v2.0, the maximum value obtained at 30 min was 171.6 AU for a KPC-producing *K. pneumoniae* and the minimum value was 11.5 for a VIM-producing *E. cloacae*. The mean of the signal values was 97.3 AU (Table 4). On the whole, 45% and 85% of CPE were detected within five minutes and 10 minutes, respectively. The results for the individual targets are presented in Table 4.

**Table 5:** Discrepant results between the BYG Carba v2.0 test and the reference method.

N°	Species	Carbapenemase	First testing			Confirmation in centre A			Additional hydrolysis testing
			BYG value at 30 min (AU)	Result	Time to pos (min)	BYG value at 30 min (AU) repeat	Time to pos (min) repeat	Result repeat	Carba NP test
1	*Enterobacter cloacae*	GES-5	0.4	FN	—	4.6	—	FN	POS
2	*Enterobacter cloacae*	GES-5	2.9	FN	—	−4.3	—	FN	POS
3	*Klebsiella oxytoca*	GES-5	−1.3	FN	-	0.8	—	FN	NEG
**4**	***Klebsiella oxytoca***	**GES-5**	**−0.5**	**FN**	—	**12.3**	**28**	**TP**	**POS**
**5**	***Enterobacter cloacae***	**IMI-1**	**−5.0**	**FN**	—	**94.0**	**6**	**TP**	**POS**
6	*Enterobacter cloacae*	IMI-2	11.0	FN	—	0.4	—	FN	NEG
7	*Escherichia coli*	IMP-1	2.0	FN	—	−0.2	—	FN	POS
**8**	***Enterobacter cloacae***	**IMP-1**	**6.1**	**FN**	—	**12.1**	**27**	**TP**	**NEG**
9	*Providencia stuartii*	NDM-1	1.9	FN	—	−1.6	—	FN	NEG
10	*Providencia rettgeri*	NDM-1	9.3	FN	—	11.4	—	FN	NEG
11	*Klebsiella oxytoca*	NDM-1 *	8.4	FN	—	0.9	—	TN	NEG
**12**	***Proteus mirabilis***	**NDM-1**	**3.9**	**FN**	—	**14.9**	**24**	**TP**	**POS**
13	*Klebsiella pneumoniae*	OXA-181	3.1	FN	—	3.5	—	FN	NEG
14	*Escherichia coli*	OXA-244	0.9	FN	—	2.2	—	FN	NEG
15	*Escherichia coli*	OXA-244	1.2	FN	—	3.1	—	FN	NEG
**16**	***Escherichia coli***	**OXA-244**	**3.7**	**FN**	—	**16.3**	**24**	**TP**	**POS**
17	*Escherichia coli*	OXA-48	9.4	FN	—	8.8	—	FN	POS
18	*Enterobacter cloacae*	VIM-1	6.8	FN	—	6.0	—	FN	NEG
**19**	***Providencia stuartii***	**NDM**	**−0.1**	**FN**	—	**32.5**	**16**	**TP**	**NEG**
20	*Klebsiella oxytoca*	OXA-232	−4.1	FN	—	1.2	—	FN	POS
21	*Escherichia coli*	OXA-244	1.1	FN	—	0.8	—	FN	POS
**22**	***Escherichia coli***	**OXA-48**	**3.6**	**FN**	—	**71.4**	**6**	**TP**	**POS**
23	*Escherichia coli*	OXA-48 *	0.2	FN	—	−1.1	—	TN	NEG
24	*Klebsiella pneumoniae*	OXA-48	10.3	FN	—	10.0	—	FN	NEG
25	*Enterobacter cloacae*	VIM-1	8.0	FN	—	9.1	—	FN	POS
26	*Escherichia coli*	VIM-4	1.1	FN	—	8.2	—	FN	POS
**27**	***Enterobacter cloacae***	**No Carba (SHV-12)**	**15.8**	**FP**	**20**	**1.1 and 4.1**	—	**TN**	**NEG**

Isolates 1 to 18 were retrospectively collected and the isolates 19 to 27 are prospectively collected.

The Carba NP imipenem hydrolysis test was performed to confirm the imipenem hydrolysis in these isolates.

FN: False negative; TP: True positive; TN: True negative; POS: positive; NEG: negative.

*These isolates have probably lost their plasmid.

Correct results obtained in centre A are in bold and underlined.

## Discussion

The accurate and timely detection of CPE is of paramount importance for prevention and control of outbreaks in clinical settings and for the management of patients infected with CPE^7^. The detection of CPE can be performed by culture methods on specific media followed by confirmatory phenotypic testing or by in-house/commercial molecular testing. Molecular methods present the advantage of rapidity and can be used for non-culture based detection of carbapenemases directly from screening samples^8^. However, a major limitation is that only the genes and allelic variants targeted by the assays can be detected. These methods are also usually expensive, necessitating specific costly instruments, or are not accessible on a routine daily basis for general clinical laboratories that lack specifically trained personnel and dedicated rooms.

Here we present the results of a multicentre evaluation of the performance of a simplified version of the recently published^5^ BYG Carba test in four laboratories in Europe. Overall, only 2.3% of discrepant results were observed in comparison to the reference molecular methods. Contrarily to molecular methods, as it is the case with colorimetric assays, the BYG carba test would be able to detect even unknown (novel) carbapenemases. One definite advantage of the BYG Carba test over the colorimetric assays^9–18^ relates to the fact that once a cut-off limit is set, the interpretation becomes objective by reporting a number (arbitrary value expressing the polyaniline conductance) and indicating the positivity in real-time.

Some metallo-β-lactamases such as IMP were detected with a lower efficiency by the BYG Carba v2.0 (86.7% sensitivity in the retrospective survey). In the prospective phase of this evaluation, only two IMP-producers were collected during the study period, confirming the extremely low prevalence of this carbapenemase in the UK, France and Belgium as already observed in Europe^19^. The performance of the BYG Carba v2.0 test against this particular carbapenemase should be further evaluated in countries with higher endemic settings, such as Japan, Korea or Taiwan. On the other hand, the BYG Carba v2.0 detected 349/359 (97.2%) of the OXA-48 like producing isolates, 90% of these (311/349) being detected in 10 minutes or less. Nevertheless, some OXA-48 variants such as OXA-244, a weak and rare carbapenemase^20^, were less well detected than other OXA-48-like allelic variants (four false negative results among five OXA-244-producers). The latter results match with those previously observed with the RAPIDEC^®^ CARBA NP^9^. Accordingly, microbiologists should be alerted to this fact and any suspicion of an OXA-48-like carbapenemase based on *in vitro* resistance to temocillin and to piperacillin/tazobactam could be confirmed directly by nucleic acid amplification methods or by the immunochromatographic OXA-48 K-SeT assay (Coris BioConcept, Gembloux, Belgium)^21, 22^. Interestingly, Meunier *et al*. reported that two GES-5-producing strains that failed to be detected with the BYG Carba assay were correctly detected by the RAPIDEC^®^ CARBA NP^23^. Again, GES-type carbapenemases very rarely occurred as shown by the fact that no GES-positive isolate was identified during the prospective evaluation.

The procedure used with BYG Carba v2.0 is very simple (supplementary video). Among commercial tests only the β-CARBA^™^ Test from Bio-Rad offers similar advantages with minimal hands-on time and low bacterial inoculum but detection remains based on visual detection of a colour change^4^. In the laboratory, the cost for a BYG Carba test prototype seems affordable. The home-made reader, adaptable to any computer, is built at a cost of 100 euros and all materials/reagents needed for the electrodes including the different coatings cost less than 1 euro per strain. It opens the possibility of industrialisation, commercialisation or even open accessibility at a price allowing its broad usage for the detection of carbapenemases. Based on the measurement of the modification of pH and redox potential by several electrodes connected in parallel, it would also permit analysis of the effect of several antimicrobials on a single isolate. Finally, after disinfection with non alcoholic chlorhexidine, we experimented that the electrodes could be reusable. But this should be further investigated.

In conclusion, the BYG Carba v2.0 is a fast and accurate test for the detection of CPE. It allows the objective detection of CPE from 1–3 colonies in less than 30 minutes and could represent a major additional tool in the laboratory armamentarium for detection of CPE. In addition the technology is very adaptable for miniaturisation and further multiplexing, permitting the testing of several antimicrobials in parallel.

## Methods

### Electrochemical instrumentation and the electrodes

The principle and technology was described in details in previous publications^24, 25^. Briefly, the system is composed of a small homemade electronic device (potentiostat), and of disposable electrodes coated with polyaniline as a sensor^5^. Up to five potentiostats can be connected at the same time to a computer through a USB Hub (serial port) allowing the simultaneous analysis of 20 isolates.

The results of the BYG Carba test are displayed as curves visualized in real-time. One curve corresponds to the signal (conductance) detected with imipenem and another curve to the signal without imipenem (background curve). The software then subtracts the background signal from the conductance signal obtained with imipenem. For non-carbapenemase-producing isolates, the resulting signal may be negative when the conductance signal obtained at 30 min with imipenem is lower than the background signal without imipenem. An isolate is reported as positive when the resulting curve crosses the cut-off. At the end of the run, the software generates a report.

### BYG Carba test v1.0 and v2.0

For the BYG Carba v1.0, the bacterial suspensions were prepared as previously described^5^.

The BYG Carba test v2.0 was recently developed and validated in order to simplify the procedure by avoiding multiple pipetting steps and by allowing the test to be performed directly from a smaller amount of bacterial growth (4, Supplementary video).

In this new protocol, the bacterial inoculum consists of one to three colonies ( + /−10^6^ CFU/ml) directly smeared onto two adjacent probes of the BYG Carba test in such a way that the sensor is completely covered by the colonies. The electrode is then overlaid with 50 µl of a 4 M NaCl; 0.3 mM ZnSO_4_ solution with or without imipenem (same concentration as in BYG Carba v1.0). The BYG Carba test measures the conductivity of the polyaniline sensor during the hydrolysis of imipenem. This signal is transformed into an objective value calculated in real-time. Once the threshold is reached, the software automatically reports the CPE positivity status of the isolate (Supplementary video). A cut-off of 11.5 (arbitrary units [AU]) was defined previously as the threshold for discrimination between carbapenemase and non-carbapenemase producers^4^.

### Bacterial isolates and characterisation

The BYG Carba v2.0 was initially validated retrospectively against a collection of 57 reference strains of Enterobactericeae including 41 CPE isolates (OXA-48-like [n = 12], KPC [n = 8], NDM [n = 8], VIM [n = 8], IMP [n = 3], GIM [n = 1] and GES-6 [n = 1]) and 16 non-CPE isolates also used for the validation of the BYG Carba test v1.0 in order to compare the signals obtained with both protocols^5^. All 57 isolates were analyzed in triplicate with both methods run in parallel.

The evaluation of the BYG Carba v2.0 was performed in each laboratory both retrospectively, on a collection of strains reflecting the local epidemiology of carbapenemases in different geographical areas in Europe, and prospectively on consecutive non-duplicate isolates referred on a voluntary basis to the national reference centres for investigation of the mechanism(s) of carbapenem resistance. Non-susceptibility to carbapenems was assessed by the reference laboratories following the European Committee on Antimicrobial Susceptibility Testing (EUCAST) guidelines (http://www.eucast.org, latest accessed August 23^rd^ 2016). Molecular testing for the detection of carbapenemases was considered as the gold standard and was performed in each laboratory as follows:

In the CHU UCL NAMUR (Yvoir, Belgium) (centre A), the BYG Carba v2.0 was initially challenged retrospectively against 150 clinical consecutive non-duplicate isolates received between January and March 2014. Subsequently, the BYG Carba v2.0 was evaluated against 258 consecutive non-duplicate Enterobacteriaceae isolates referred between August and November 2015. The isolates were characterized as previously described^5^.

Carbapenemases were sought by two in-house ISO15189-certified multiplexed PCRs targeting *bla*_OXA-48-like_, *bla*_NDM_, *bla*_KPC_, *bla*_VIM_ and *bla*_IMP_^26^, and the amplicons were sequenced using external Sanger sequencing services (Macrogen, Seoul, Korea) for allele identification.

In the ULB Erasme hospital associated national reference centre (Brussels, Belgium) (centre B), 86 clinical retrospective isolates representative of the hospital epidemiology received between January 2014 and October 2015, and 112 prospective isolates received between November 2015 and April 2016 were analyzed. These isolates were characterised according to the same procedures as in centre A.

In France (centre C), the BYG Carba v2.0 was challenged retrospectively against a collection of 175 Enterobacteriaceae already used for the validation of other carbapenemase diagnostic tests^9^ and prospectively against consecutive 201 non-duplicate clinical enterobacterial isolates received at the French National Reference Centre for Antibiotic Resistance between 15^th^ February and 15^th^ March 2015 for carbapenemase identification. These isolates were analyzed according to the procedure published previously^27^.

In Public Health England’s Antimicrobial Resistance and Healthcare Associated Infections (AMRHAI) Reference Unit (London, UK) (centre D), the BYG Carba v2.0 was retrospectively evaluated on 99 Enterobacteriaceae representative of the UK epidemiology and received between January 2013 and December 2015 and prospectively on 100 consecutive Enterobacteriaceae isolates received between April and July 2015. Carbapenemase genes were sought using in-house multiplex PCRs^28, 29^ and confirmed by WGS using a Hiseq sequencer (Illumina) for the retrospective isolates. The resulting WGS data were analyzed using an in-house bioinformatics pipeline with resistance genes identified by mapping reads to an in-house library curated from publicly-available databases^30^.

Any discrepancies between the BYG Carba v2.0 and molecular assay results obtained in centres B, C, D, were reanalyzed in centre A following its own procedure for bacterial identification, antibiotic susceptibility testing by disc diffusion and PCR for carbapenemase genes. The isolates were also retested with the BYG test v2.0 in centre A. The results subsequently obtained in centre A, were not used for the calculation of the performance of the test but only to investigate the potential sources of discrepancies. Sensitivity, specificity, positive and negative predictive values were calculated using the free software vassarStats: Website for statistical Computation on http://vassarstats.net/.

## Electronic supplementary material

BYG carba v2.0 video

BYG carba v2.0 video legend
