# Supplementary material for: Multicentre evaluation of the BYG Carba v2.0 test, a simplified electrochemical assay for the rapid laboratory detection of carbapenemase-producing Enterobacteriaceae
Source: Sci Rep. 2017 Aug 30;7:9937. doi: 10.1038/s41598-017-09820-y (PMC5577128; doi:10.1038/s41598-017-09820-y)
Supplement: Supplementary file 2 — BYG carba v2.0 video legend [file 41598_2017_9820_MOESM2_ESM.doc]

Supplementary BYG carba v2.0 video legend

Multicentre evaluation of the BYG Carba v2.0 test, a simplified electrochemical assay for the rapid laboratory detection of carbapenemase-producing Enterobacteriaceae

Pierre Bogaerts^1*^, Saoussen Oueslati^2,3,4+^, Danièle Meunier^5+^, Claire Nonhoff^6+^ , Sami Yunus^7^ , Marion Massart^1^, Olivier Denis^6^, Neil Woodford^5^, Katie L Hopkins^5^, Thierry Naas^2,3,4^, Laurent Dortet^2,3,4^, Te-Din Huang^1^, Youri Glupczynski^1^

The video presents the use of the BYG carba v2.0 in routine.

The disposable electrode comprises eight probes and is plugged in an electronic device connected to a computer. Each isolate is loaded on two probes (probe - and probe +) on the working electrode. One probe is recovered with the buffer without imipenem (probe -) to measure the background and one probe is recovered with the buffer with imipenem (probe +) to measure the hydrolysis of imipenem.

The video shows:

- the loading of the bacterial colonies, including a positive control (OXA-48 producing isolates), on each of the eight probes,
- the loading of the buffer on the electrode,
- the starting up of the experiment,
- the signals (curve) obtained by the instrument during the test
- the interpretation of the results by the software
  - As soon as the curve crosses the treshold, the cell including the name of the isolate is coloured in red by the software indicating a carbapenemase producer. At the end of the run, non carbapenemase strain’s cell is coloured in green.
  - A report is finally created as a pdf file
